# Transcranial static magnetic field stimulation over hMT+ inhibits visual motion discriminability

**DOI:** 10.1038/s41598-023-51097-x

**Published:** 2024-01-11

**Authors:** Ayaka Takami, Toshitaka Kawajiri, Takaaki Komiyama, Chisa Aoyama, Satoshi Shimegi

**Affiliations:** 1 Graduate School of Frontier Biosciences, Osaka University, Toyonaka, Osaka Japan; 2 Present Address: Center for Education in Liberal Arts and Sciences, Osaka University, Toyonaka, Osaka Japan; 3 Graduate School of Medicine, Osaka University, Toyonaka, Osaka Japan

**Keywords:** Neuroscience, Psychology

## Abstract

Visuomotor performance acting on a moving target is fundamentally based on visual motion discriminability, and its neural basis is presumed to be human MT (hMT+), a motion vision center of the dorsal visual pathway. In this study, we investigated whether and how the accuracy and speed of motion discrimination are affected by applying transcranial static magnetic field stimulation (tSMS) to hMT+, which reduces cortical excitability. Sixteen participants performed a motion direction discrimination (MDD) task using a random dot kinematogram before (Pre-test) and during (During-test) application of the tSMS over left hMT+. The correct rate of the MDD task was significantly lower in the During-test compared to the Pre-test, an effect not seen with the sham condition. The inhibition effects were observed only for the right visual field corresponding to hMT+ in the stimulated hemisphere. On the other hand, no modulatory effect of tSMS was observed in the reaction time. We, therefore, demonstrated the inhibitory effect of tSMS on the left hMT+ impairs the accuracy but not the speed of motion information processing in the contralateral visual field.

## Introduction

Visuomotor performance in ball sports like table tennis depends on not only physical^1,2^ but also neural factors especially the visual system processing of the ball’s motion information^3^. Visual information input to the retina is transmitted to the primary visual cortex via the lateral geniculate nucleus and then processed in parallel by the ventral and dorsal visual pathways^4^. The ventral pathway contributes to the processing of color and shape. On the other hand, the dorsal pathway processes information, such as the position and motion of objects, and contributes to the execution of bodily actions toward the outside world, such as reaching and grasping for objects. Therefore, the dorsal pathway plays an important role in hitting and catching a ball and is considered to be an important determinant of visuomotor performance^3^.

The dorsal pathway includes the human V5/MT+ complex (hMT+, the putative homolog of macaque MT), which is the center for processing visual motion and generating visual motion sensation/perception^5–7^. hMT+ is located in the posterior bank of the superior temporal sulcus of the dorsal medial temporal cortex and the parietal cortex^8,9^. The various interventions such as transcranial direct current stimulation (tDCS)^10^ and repetitive transcranial magnetic stimulation (rTMS)^11^ over hMT+ indicate that the external noninvasive interventions on the excitability of hMT+ modulate the accuracy of visual motion discrimination but it has been unclear whether the speed (reaction time) is modulated.

Transcranial static magnetic field stimulation (tSMS)^12^ is a new brain stimulation method that uses a high-powered cylindrical neodymium, iron, and boron (NdFeB) magnet, which reduces the excitability of the cerebral cortex locally, noninvasively, and safely^13^. tSMS has been reported to decrease the excitability of the human motor area^12,14–17^, human somatosensory area^18,19^, and monkey primary visual area^20^. Moreover, the application of tSMS over the primary visual cortex induced behavioral changes such as a reduction of contrast sensitivity and an extension of reaction time in visual detection task^21^ and visual search task^22^. However, no studies examined the effects of tSMS on hMT+. Therefore, it remains unknown whether and how the accuracy and reaction time of the visual motion discrimination are impaired individually. A previous study demonstrated that the application of the rTMS over left hMT+ inhibited visual motion discriminability in the right visual field^11^. This last finding is consistent with MT neurons having receptive fields in the contralateral visual field^23^, such that hMT+ in the left (right) hemisphere is mainly responsible for the right (left) visual field. Based on the above, we investigated the inhibitory effect of tSMS over the left hMT+ from the viewpoint of the accuracy and speed of perceptual decision-making for visual motion direction discrimination in the present study.

## Results

### Effects of tSMS/sham stimulus intervention on the correct rate of the MDD task in contralateral (right) and ipsilateral (left) visual fields

**Figure 1:**
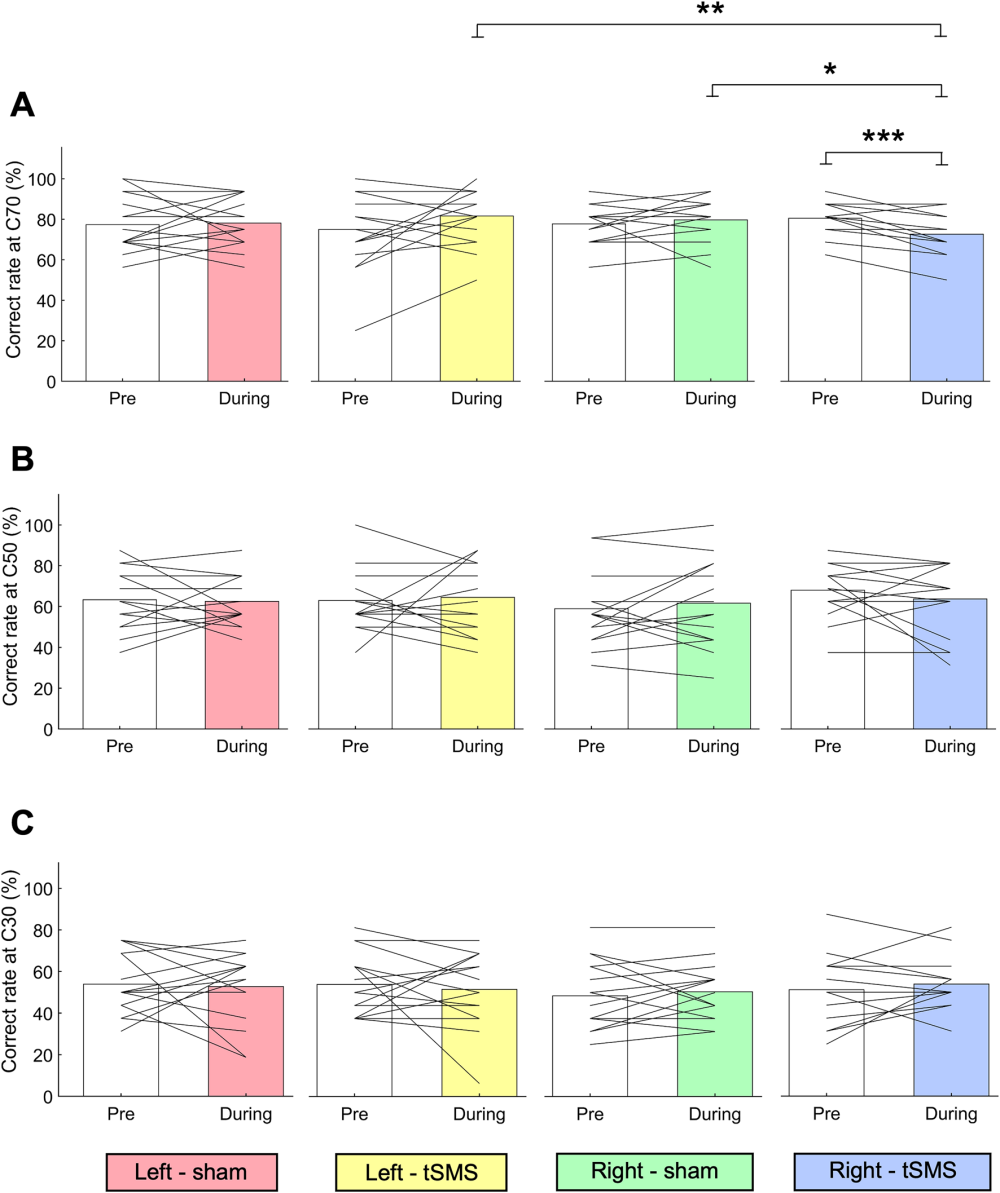
The correct rate at (**A**) C70, (**B**) C50, and (**C**) C30 in left-sham (pink), left-tSMS (yellow), right-sham (green), and right-tSMS (blue) conditions. C70, C50, and C30 represent the motion coherence of the target stimulus corresponding to each participant’s 70%, 50%, and 30% correct rate in the MDD task, respectively. *p < 0.05, **p < 0.01, ***p < 0.001.

Figure 1 shows that a significant interaction was observed at C70 which was the highest motion coherence condition (F_1,15_ = 8.721, *p* < 0.01, η*p*^2^ = 0.368).

Therefore, we compared the correct rate between the Pre-and During-tests. In the right visual field, the rate for the During-test was significantly lower than for the Pre-test with tSMS (*p* < 0.001, η*p*^2^ = 0.568) but not for sham (*p* = 0.474). The left visual field showed no significant difference for either condition (tSMS, *p* = 0.132; sham, *p* = 0.833).

Additionally, in the right visual field, the rate for the During-test was lower with tSMS than with sham (*p* < 0.05, η*p*^2^ = 0.327), but no significant difference was observed for the Pre-test (*p* = 0.168). In the left visual field, there was no significant difference between either test (Pre-test, *p* = 0.648; During-test, *p* = 0.351).

Finally, for the During-test in the tSMS condition, the correct rate of the right visual field was significantly lower than that of the left visual field (*p* < 0.01, η*p*^2^ = 0.479), but the Pre-test value showed no significant difference between hemi-visual fields (*p* = 0.245).

No significant main effect or interaction was found for C50 or C30 which was the middle and lowest motion coherence condition, respectively (C50, *p* = 0.386; C30, *p* = 0.955).

### Effects of tSMS/sham stimulus on all reaction time of the MDD task in contralateral (right) and ipsilateral (left) visual fields

**Figure 2:**
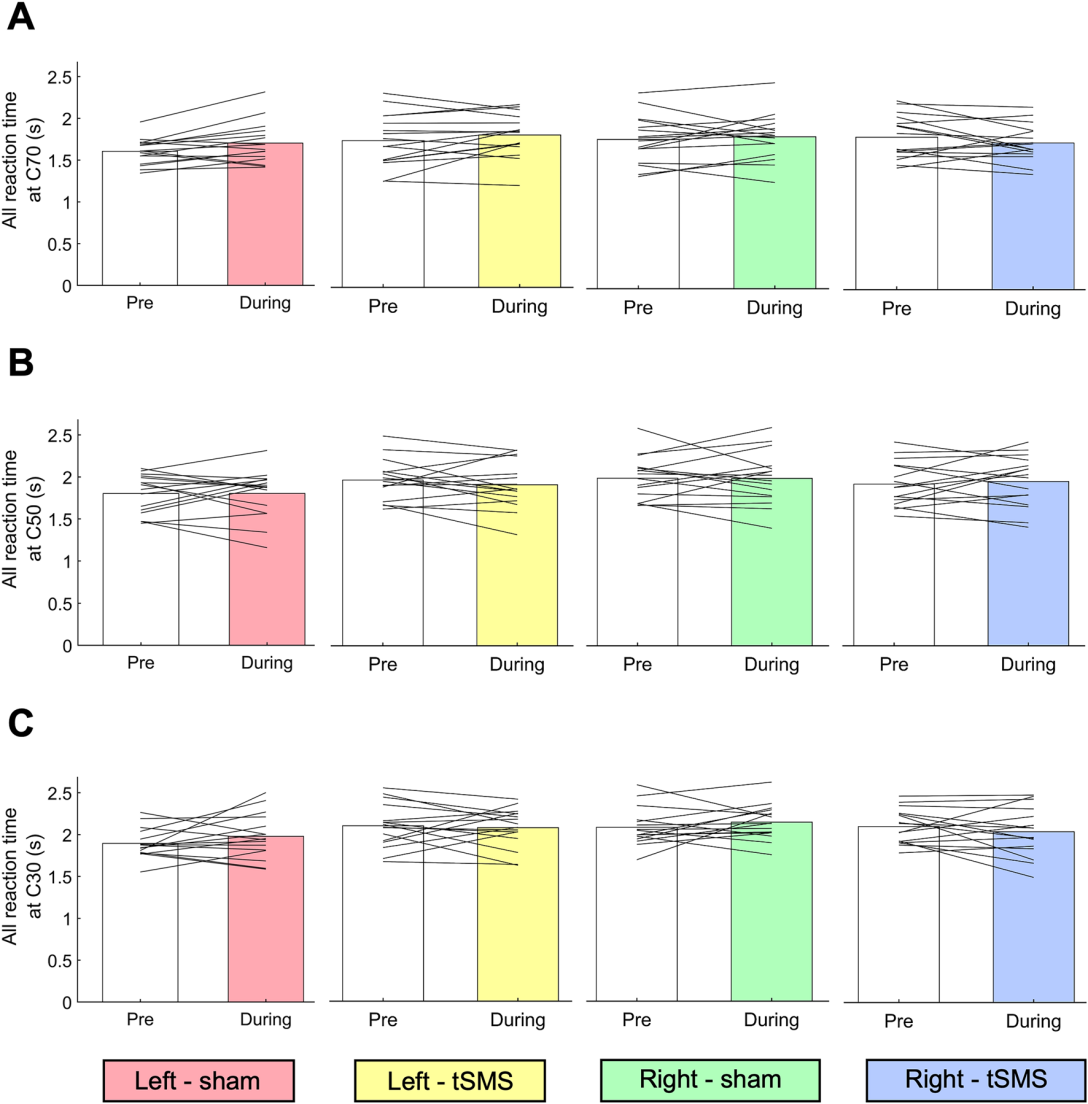
The all reaction time at (**A**) C70, (**B**) C50, and (**C**) C30 in left-sham (pink), left-tSMS (yellow), right-sham (green), and right-tSMS (blue) conditions.

Figure 2 shows the result of all reaction time. For all reaction time, there was no significant main effect (C70-Stimulus position, *p* = 0.961; C70-Stimulus type, *p* = 0.790; C70-Time, *p* = 0.211; C50-Stimulus position, *p* = 0.341; C50-Stimulus type, *p* = 0.665; C50-Time, *p* = 0.875; C30-Stimulus position, *p* = 0.723; C30-Stimulus type, *p* = 0.693; C30-Time, *p* = 0.645) or interaction (C70, *p* = 0.344; C50, *p* = 0.367; C30, *p* = 0.910) for any motion coherence.

### Effects of tSMS/sham stimulus on the correct reaction time of the MDD task in contralateral (right) and ipsilateral (left) visual fields

**Figure 3:**
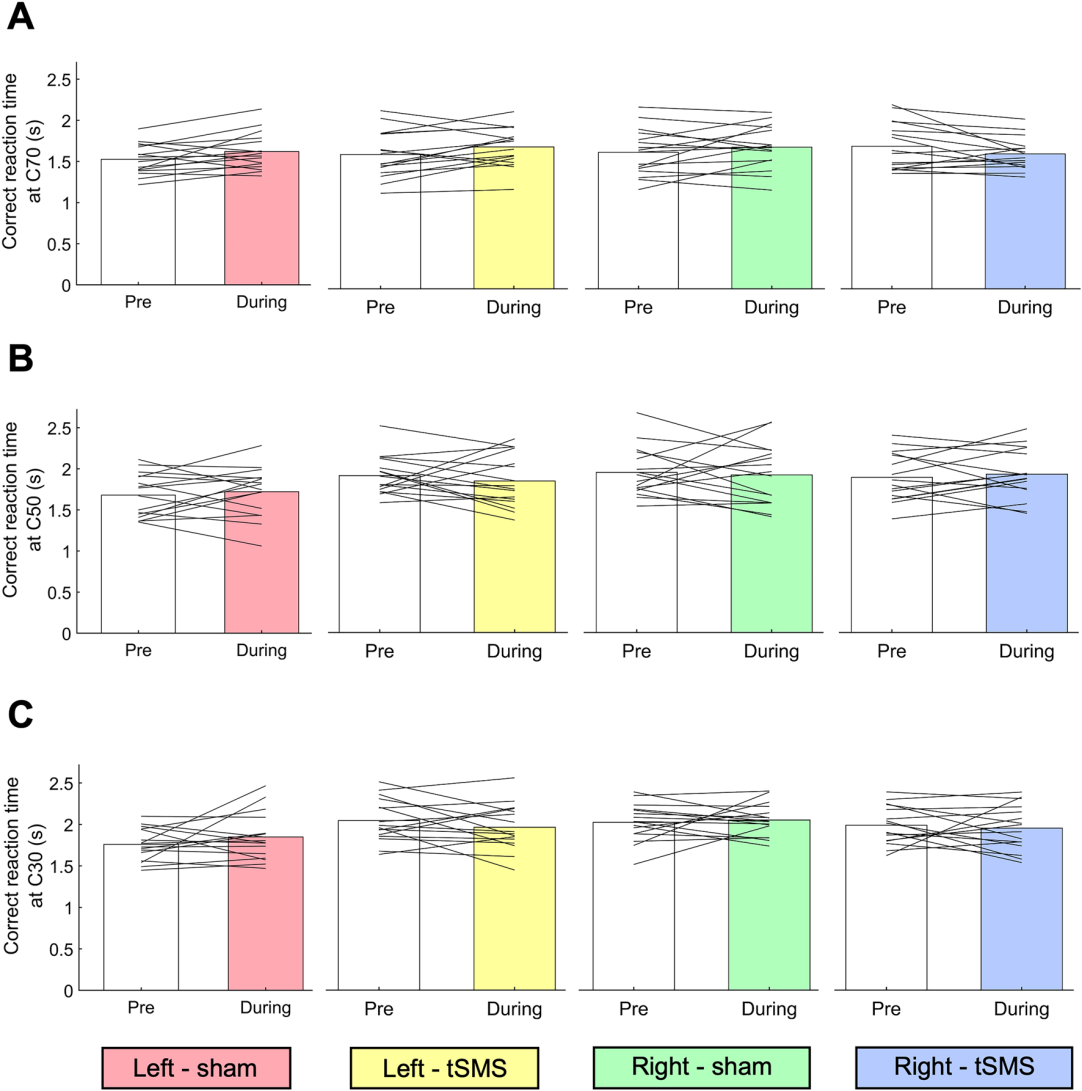
The correct reaction times at (**A**) C70, (**B**) C50, and (**C**) C30 in left-sham (pink), left-tSMS (yellow), right-sham (green), and right-tSMS (blue) conditions.

Figure 3 shows the result of the correct reaction time. For the correct reaction time, there was a significant interaction at C70 (F_1,15_ = 5.476, *p* < 0.05, η*p*^2^ = 0.267). In the ipsilateral (left) visual field, the correct reaction time of the During-test was significantly longer than that of the Pre-test regardless of the stimulus type (*p* < 0.05, η*p*^2^ = 0.321), suggesting the difference is not ascribed to the stimulus intervention. For C50 and C30, there was no significant main effect (C50-Stimulus position, *p* = 0.151; C50-Stimulus type, *p* = 0.925; C50-Time, *p* = 0.955; C30-Stimulus position, *p* = 0.685; C30-Stimulus type, *p* = 0.730; C30-Time, *p* = 0.935) or interaction (C50, *p* = 0.129; C30, *p* = 0.364).

### Identification of stimulus type of participants

We confirmed that at the end of each experimental day, participants were unable to identify the type of stimulus they were exposed to (tSMS or sham; *χ*^2^ = 0.25, *df* = 1, *p* = 0.62).

## Discussion

In this study, we examined whether and how the change of visual motion discriminability in the contralateral (right) and ipsilateral (left) visual fields when the excitability of left hMT+ was reduced by tSMS. Significant effects of tSMS were observed in the correct rate of the MDD task only in the right visual field at the C70 condition.

The main results are summarized as follows: (1) the During-test correct rate under tSMS was significantly lower than the Pre-test correct rate (*p* < 0.001), (2) it was also lower under tSMS than sham (*p* < 0.05), (3) and in the tSMS condition, the During-test correct rate of the right visual field was significantly lower than that of the left visual field (*p* < 0.01), (4) no differences were observed between the Pre-test and the During-test in either the all reaction time or the correct reaction time regardless of the tSMS/sham condition, (5) the correct reaction time for the left visual field was significantly longer in the During-test than in the Pre-test (*p* < 0.05) in both tSMS and sham conditions.

In humans, various visual areas, including hMT+, are involved in visual motion perception^24^. hMT+ is thought to be a homologue of MT of the primate macaque monkey, and damage to MT inhibits visual motion discriminability^5,6^. In addition, neuronal activity of MT correlates highly with discrimination sensitivity and with trial-to-trial fluctuations in the motion direction discrimination task using coherent motion produced by random dot kinematograms (RDK) like those used in the present study^25^. Moreover, interventions in hMT+ using various noninvasive brain stimulations have been reported to affect visual motion discriminability, supporting a causal relationship between neuronal activity in hMT+ and visual motion discriminability. For example, applying rTMS over hMT+ inhibits cortical excitability, lowering the discriminability of motion speed^26^ and motion direction^11^. Additionally, the application of tDCS over hMT+ enhances motion perception^10^. Considering that tSMS reduces the excitability of the cerebral cortex locally, these studies and the present study suggest that reduction of the excitability of hMT+ impairs the performance of visual motion discriminability.

In the present study, applying tSMS over left hMT+ significantly lowered the correct rate of the MDD task in the right (contralateral) visual field (*p* < 0.001) but not in the left (ipsilateral) visual field. This result agrees with a previous study using rTMS^11^. The application of rTMS over left hMT+ lowered the correct rate only in the right visual field of the motion direction discrimination task using RDK. The reduced excitability of left hMT+ inhibited motion direction discriminability for the contralateral visual field. However, applying rTMS over right hMT+ attenuated visual motion discriminability not only in the left (contralateral) but also in the right (ipsilateral) visual field. Thus, the effects of unilateral cortical intervention on the visual field differ between the right and left hemispheres. This hemispheric difference may be attributed to differences in the receptive field properties in hMT+, in which neurons in left hMT+ have their receptive field restricted to the right visual field, but the receptive field of right hMT+ neurons cover not only the contralateral visual field but also part of the ipsilateral visual field. Therefore, the application of tSMS over right hMT+ may inhibit the motion direction discriminability of both visual fields. Future research is needed to clarify this point.

Not only the accuracy but also the speed of perceptual judgments has been known to depend on the strength of the sensory stimulus, and lower stimulus intensities result in lower accuracy and slower reaction times. Consistent with this, we also observed that a decrease in motion coherence corresponding to stimulus intensity also increased reaction time. Since tSMS reduces cortical excitability and reduces responses to sensory stimuli of the same intensity, similar to when lower-intensity sensory stimuli are presented, reaction times may also increase. In the present study, tSMS over hMT+ impaired the accuracy of motion direction discriminability but not the speed measured as the reaction time. On the other hand, the application of the tSMS over the primary visual cortex in monkeys performing the stimulus detection task using uniform patch stimuli with different contrasts has been reported to not only decrease the correct rate but also extend the reaction time. A possible reason for the discrepancy between the study and ours is whether there is a time limit for responding. The present study set a time limit of 3 s, in which the participants had to answer within the limit even if their perception was obscure. On the other hand, the monkeys were not restricted by the time limit and could respond after monkeys reached perceptual decision-making through the accumulation of the target information.

In this study, in the left visual field, regardless of whether the stimulus type was sham or tSMS, the correct reaction time of the During-test was significantly longer than that of the Pre-test. The reason for the longer correct reaction time due to the time passage could be the accumulation of fatigue or a decrease in concentration. It has been known that attentional modulation on visual perception is not even for hemi-visual fields and is stronger for the left visual field^27^. Therefore, the change in reaction time through the time lapsed in the experiment might result from attentional fatigue and distraction.

Applying tSMS during a visual stimulus detection task over the primary visual area of cats and monkeys impaired contrast sensitivity by suppressing neural activity^21^. To date, several action mechanisms of tSMS on the cerebral cortex have been proposed. In one theory, tSMS rotates and rearranges phospholipids in the cell membrane, thereby deforming the ion channels and altering their activity speeds^28–31^. Another possibility has been proposed recently that tSMS induces magnetic pressure which may contribute to the long-lasting effects of the tSMS over the cortex by interfering with elastic and electrostatic energies involved in the channel activation-inactivation-deactivation mechanisms of biological membranes. A small mechanical force can activate voltage-gated potassium channels, hyperpolarizing cortical neurons^32^.

To discriminate the motion direction of the target in the MDD task, participants need to separate the target moving in the same direction (motion signals) from the non-target moving in random directions (noise). Therefore, the greater the intensity of the motion signals compared to the noise, the easier the signals are separated from the noise^33^. It has been reported that neural activity of hMT+ in response to the motion signal is stronger than in response to noise^34^, and increasing the motion coherence of the target enhances the neural activity of hMT+^35,36^. In the present study, regardless of the stimulus position or stimulus type, the correct rate in the MDD task at the Pre-test was higher in the order of C70, C50, and C30 (*p* < 0.01). In low coherence conditions, such as C30, the signal intensity is low, making separation of the signal from noise difficult, which may explain the reduced visual motion discriminability. On the other hand, in high coherence conditions, such as C70, the strong signal intensity simplifies the separation, which may explain the enhanced visual motion discriminability. Therefore, the correct rate in this study likely reflects the difficulty of separating the signal from noise.

The effect of tSMS on visual motion discriminability was demonstrated at only C70 of the three studied motion coherence conditions (C30, C50, and C70). As mentioned above, motion coherence is equivalent to the intensity of the visual stimulus input. Consistently, the effect of tSMS on the detectability of visual stimuli has been reported to differ depending on the stimulus contrast (the intensity of the visual stimulus)^21^. In that study, tSMS for 30 min applied over the primary visual cortex of monkeys reduced the stimulus detection rate at only the high contrast condition. Thus, the tSMS effect strongly depends on the visual input intensity, and the effect becomes more evident at higher input intensities, as we show with C70. Further research is needed to elucidate the mechanism with which the visual stimulus intensity causes different tSMS effects.

## Methods

### Participants and ethical approval

16 table tennis players (mean ± SD: age = 21.1 ± 1.8 years; table tennis experience = 8.6 ± 3.0 years; 6 females; 1 left-handed) met the following requirements to participate in this study: (1) normal or corrected-to-normal visual acuity; (2) no pacemakers or defibrillators in the heart; (3) no allergy to nickel; and (4) no metal implants, including clips, coils, ventriculoperitoneal shunt, prostheses, or hearing aids on the body. All participants were in good shape on the experimental day. The protocol was approved by the ethics committee of the Graduate School of Medicine, Osaka University (L021), and was conformed in accordance with the Declaration of Helsinki. Each participant provided written informed consent.

### Motion direction discrimination (MDD) task

**Figure 4:**
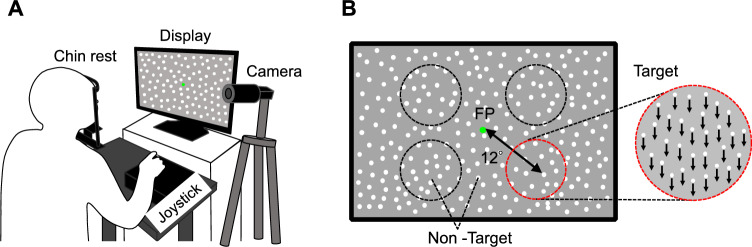
Schema of the MDD task setup. (**A**) The participant put his/her head on a chin rest and responded to moving dots by tilting a joystick. (**B**) The target stimulus was presented at any one of four circular areas that were set 12° away from the fixation point (FP). In the shown case, the target presents 315° visual angles diagonally from the FP, and the motion direction of the target is downward.

We used the MDD task^3^ to evaluate visual motion discriminability. In this task, visual stimuli were generated using a custom-made program in Python and displayed on a liquid crystal (LC) display (Iiyama, Tokyo, Japan; resolution, 1920 × 1080 pixels; refresh rate, 100 Hz; mean background luminance, 30 cd/m^2^; screen size, 60° × 34° at a viewing distance of 57 cm). Participants sat about 57 cm away from the LC display. To restrict their head movement, their heads were fixed on a chinrest (TKD-UK1, Namoto Trading Co., Ltd., Chiba, Japan) that was positioned at the center of the LC display. Participants used a joystick (JC-AS01BK, Elecom, Osaka, Japan) to respond to a visual stimulus. During the task, the participant’s right eye movements were recorded using a USB camera (Grasshopper3, Point Gray, Japan) and an eye-tracking system [iRecHS2^37^] at 500 Hz (Fig. 4A). As the visual stimulus, moving dots were presented on the LC display (Fig. 4B). The diameter of each was 0.1°, the lifetime was 180 ms (18 frames), the density was 1.5 dots/deg and the speed was 15 deg/s. The visual stimulus consisted of target and non-target stimuli and a fixation point (FP) presented at the center of the LC display. The target stimulus contained dots moving in the same direction within a circular area (8° visual angle in diameter). Within the target stimulus, the ratio of dots moving in the same direction to all dots was defined as the motion coherence. The moving direction of the target stimulus was either upward, downward, rightward, or leftward and changed randomly trial by trial. The location of the target stimulus was any one of four circular areas (45°, 135°, 225°, or 315° visual angles counterclockwise from the upper right) that were set 12° away from the FP. The target stimulus located randomly changed to one of these four positions for each trial, and the whole display outside the target stimulus displayed dot stimuli (non-target stimulus) that moved in random directions with 0% motion coherence.

**Figure 5:**
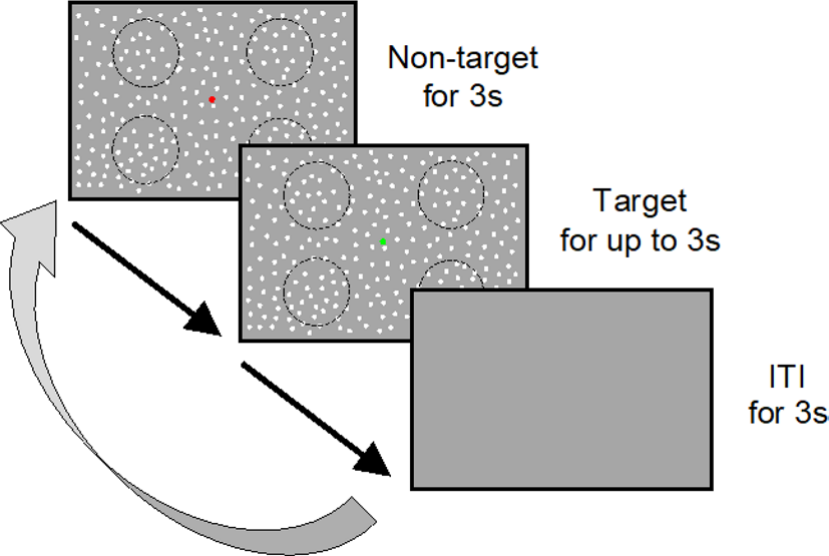
A series of one trial of the MDD task. The non-target was presented for 3 s with a red FP, and the target was presented when the color of the FP turned green. After a response by the subject or 3 s (whichever was first), all stimuli on the display disappeared for 3 s and then the next trial began. ITI stands for inter-trial interval.

The task started by presenting non-targeted stimuli on the whole display concurrently with a red FP (Fig. 5). After 3 s, the color of the FP’s color turned green, and the target stimulus was presented in any one of four circular areas and maintained for up to 3 s if the participant did not respond. Four dotted circles are shown here to indicate the stimulus presentation location, but they were not displayed in the actual task, only the moving dots are displayed. Then, all stimuli on the display disappeared for 3 s (ITI; intertrial interval), and after another 3 s, the next trial started. Participants were instructed not to move their eyes, to keep gazing at the FP during the task and to indicate the motion direction of the dots in the target by tilting the joystick toward the discriminated direction as quickly as possible with the dominant hand after the FP’s color turned from red to green. Trials in which participants tilted the joystick toward the correct direction were defined as a “correct trial,” and tilting the joystick toward the incorrect direction or no response in 3 s was defined as an “incorrect trial.” When participants responded, feedback was given by sound.

### Experimental protocol

**Figure 6:**
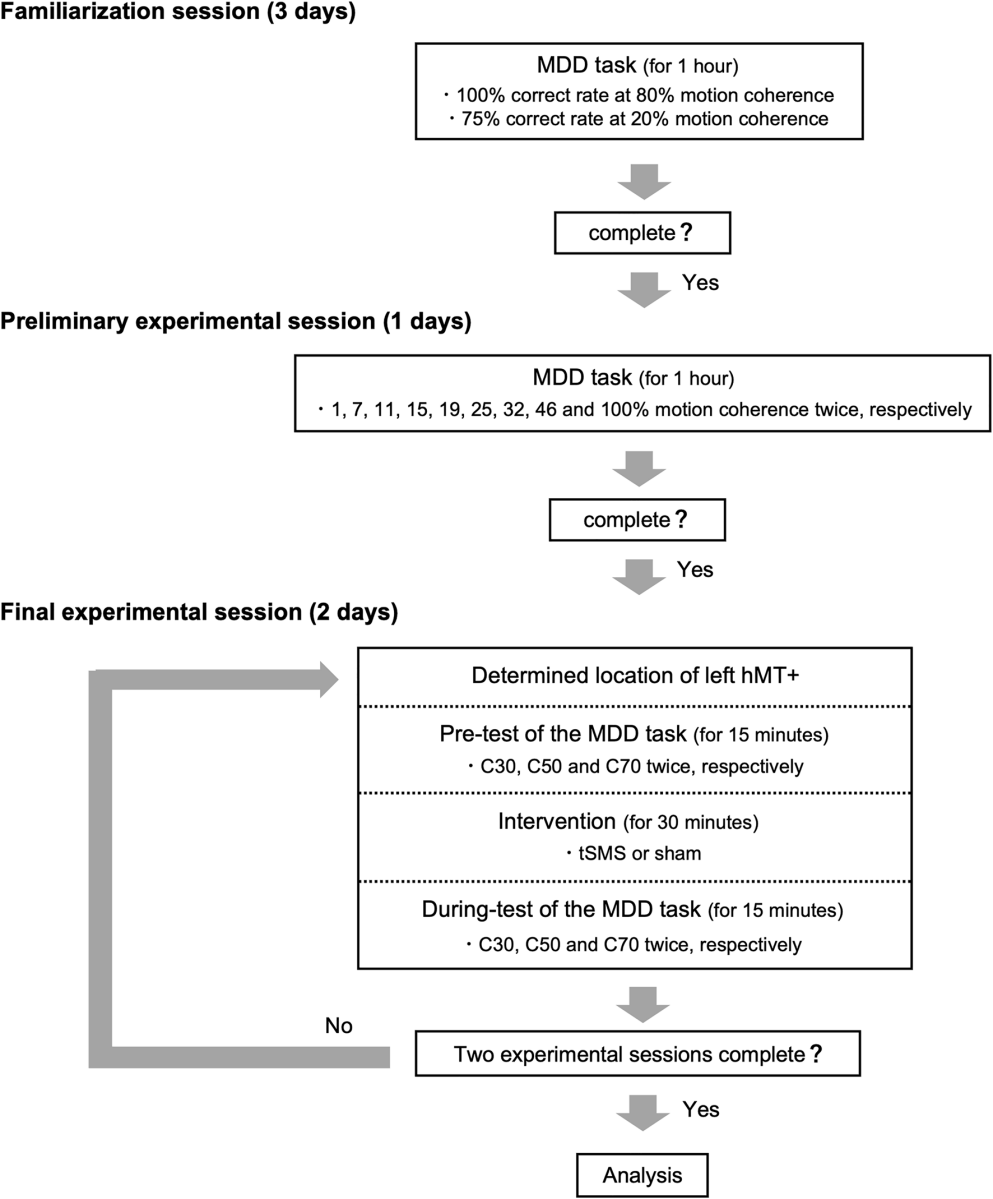
Schematic overview of the experimental protocol. The present study was composed of a familiarization session, a preliminary experimental session, and a final experimental session.

Participants performed the MDD task multiple times; thus, there was a need to exclude the effect of adaptive learning induced by the repetition of the tasks from the task performance. Moreover, the MDD task should be conducted at the same level of difficulty across individuals, but visual motion discriminability differs between individuals. For these reasons, this study consisted of a familiarization session (3 days), a preliminary experimental session (1 day), and a final experimental session (2 days) (Fig. 6).

In the familiarization session, participants performed the MDD task until the ratio of correct trials to total number of trials (correct rate) attained 100% and 75% for motion coherences of 80% and 20%, respectively.

**Figure 7:**
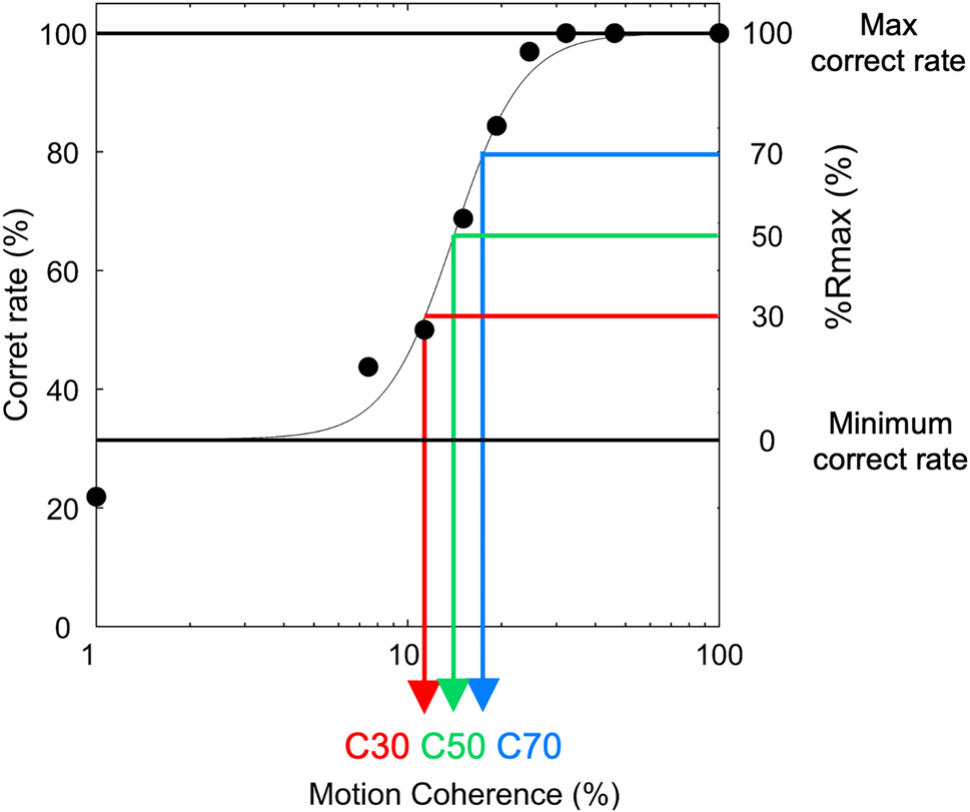
Typical result of a preliminary experiment. Participants performed the task at 9 motion coherences (black-filled circles), and the correct rate was fitted to a sigmoid curve. The difference between the maximum correct rate and the minimum correct rate was set to 100% (Rmax), and motion coherence values of 30, 50, and 70% Rmax were calculated and defined as C30 (red), C50 (green), and C70 (blue), respectively.

In the preliminary experimental session, participants performed the MDD task at 9 motion coherence conditions (1, 7, 11, 15, 19, 25, 32, 46, and 100%) twice with a rest period of about 5 min. The total number of trials for each condition was 32 trials. Figure 7 shows a typical result of the preliminary experiment. The average correct rate (black-filled circle) was calculated, and the values were fitted to a sigmoid curve using the Naka–Rushton function^38^. The difference between the maximum and minimum correct rates was set to 100% (Rmax). We also calculated 30, 50, and 70% Rmax, which were defined as C30, C50, and C70, respectively. These motion coherence conditions were used in the final experimental session to avoid ceiling/floor effects caused by too-easy/too-difficult task conditions. The time of the preliminary experimental session was 1 h.

**Figure 8:**
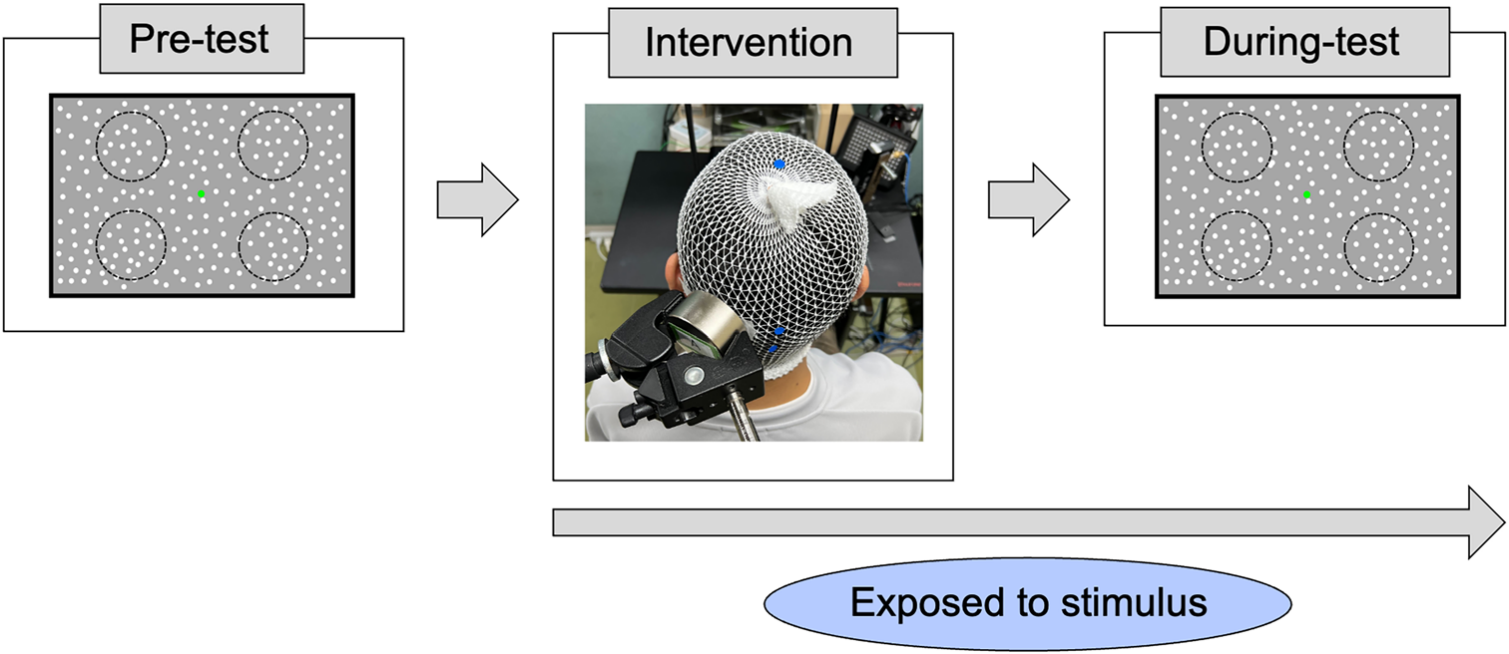
Experimental protocol of the final experimental session. Participants performed the MDD task (Pre-test), were exposed to a stimulus (tSMS or sham) for 30 min (Intervention), and performed the MDD task while being exposed to the stimulus (During-test).

The final experimental session was composed of 2 days. On the first day, participants wore stretch net bandages on their heads, and the locations of left hMT+ and the target of the stimulation were determined using previous studies^39–46^. The site of hMT+ was 3 cm dorsal to the inion and 5 cm leftward from there for each participant. Then, participants performed the MDD task at the 3 motion coherence conditions (C30, C50, and C70) once (Pre-test). The number of trials for each condition was 32 trials. Next, participants were exposed to a stimulus (tSMS or sham) for 30 min over left hMT+ in sitting rest states (Intervention) (Fig. 8). We used a cylindrical neodymium (NdFeB) magnet (diameter = 60 mm, thickness = 30 mm, weight = 670 g, nominal magnetic strength = 120 kg, MAG60r, Neurek, Toledo, Spain) for tSMS, and a steel metal cylinder of the same size for the sham stimulus. It has been reported that the reduction of cortical excitability by tSMS does not depend on the magnetic field polarity^12^; therefore, the south magnetic field polarity was adopted for all participants. After 30 min of the intervention, the participants had their heads fixed with a chinrest and performed the MDD task at the 3 motion coherences once under the subsequent stimulus exposure (the During-test). Thus, participants were exposed to stimuli during the Intervention and During-test, which together lasted for approximately 40 min. At the end of the second day, participants were asked to which stimulus they were exposed (sham or tSMS)^12,14,22,47,48^. The time of the final experimental session was 90 min per day. The final experimental session was conducted in a double-blind and crossover manner. The two days for the final experimental session were scheduled to be the same hour of the day for each participant one week apart. Participants were instructed to abstain from alcohol and caffeine for 24 h before each experimental day.

The order of the motion coherence conditions of the MDD task in each session was random in order to be counterbalanced across participants. All participants took part in all sessions.

### Data and statistical analysis

Visual motion discriminability was assessed as the correct rate and reaction time in the MDD task, which was calculated for each motion coherence condition. The correct rate was calculated from the data of all trials. The mean reaction time of all trials (all reaction time) and the mean reaction time of correct trials (correct reaction time) were calculated from data except for data of timeout trials.

We investigated whether the raw data had a normal distribution by the Shapiro–Wilk test in each motion coherence condition. For data with a normal distribution, a three-way repeated-measures ANOVA [Stimulus position (Left and Right) × Stimulus type (sham and tSMS) × Time (Pre and During)] was performed for the correct rate, reaction time, and correct reaction time. When main or interaction effects were observed, a post-hoc test using multiple comparisons with the Bonferroni correction was applied, and we calculated the effect size (η*p*^2^).

For data with a non-normal distribution, we performed a three-way analysis using Friedman’s test.

We examined whether participants could identify the type of stimulation (tSMS/sham) by the Chi-square test. The significance level was set at 5%.

## Data Availability

The datasets generated during and/or analysed during the current study are available from the corresponding author on reasonable request.
